# Spatial occurrence and sources of PAHs in sediments drive the ecological and health risk of Taihu Lake in China

**DOI:** 10.1038/s41598-022-07507-7

**Published:** 2022-03-07

**Authors:** Xiulu Lang, Xinghua He, Yanhua Wang, Xi Chen, Mingli Zhang, Zihan Zhao, Tian Sun

**Affiliations:** 1 School of Geography, Nanjing Normal University, 1 Wenyuan Road, Qixia, Nanjing, 210023 China; 2 Key Laboratory of Virtual Geographic Environment, Ministry of Education, Nanjing Normal University, Nanjing, 210023 China; 3 Jiangsu Center for Collaborative Innovation in Geographical Information Resource Development and Application, Nanjing, China; 4 School of Geographical Information and Tourism, Chuzhou University, Chuzhou, 239000 China

**Keywords:** Environmental chemistry, Environmental impact, Riparian ecology

## Abstract

To study the spatial occurrence, sources, and ecological risks of 16 PAHs, surface sediments had been collected from seven major areas of Taihu Lake, China in April 2021. Results showed that the concentrations of ∑_16_PAHs varied between 1381.48 and 4682.16 ng g^−1^, and the contents of BghiP in each sample were the highest. The PAHs concentrations in the sediments near the lakeshore were much higher than those in the central area of the lake. The sedimentary ∑_16_PAHs were mainly composed of molecular-weight monomers and 4-ring PAHs showed superiority (35.69–45.02%). According to the ratio of PAH monomer, the sedimentary PAHs in Taihu Lake were dominantly derived from the combustion. Through the biological toxicity assessment and the BaP equivalent (BaPE), great biological risks of PAHs monomers i.e. DahA and IcdP were found. Both concentrations of ∑_16_PAHs and dominant 4–6-ring monomers accompanied by carcinogenic risks in many areas of Taihu Lake increased. It is necessary to strengthen monitoring and take measures to control the input of organic pollutants.

## Introduction

Polycyclic aromatic hydrocarbons (PAHs) are typical persistent organic pollutants, which have mutagens, carcinogens, teratogens, and gene toxins, and exist in different environmental media for a long time^1–3^. PAHs could be accumulated through atmospheric deposition, growth of surface vegetation in soil and plants, and then enter the aquatic environment through surface runoff, thereby affecting water ecological security and food chain transmission^4^. Besides, PAHs in the aquatic environment may be derived from fuels, incomplete combustion, bioorganic metabolism, and the transformation process in sediment^5^. Among them, fuels and incomplete combustion were anthropogenic-driven, an important contributor to PAHs pollution in aquatic ecosystems.

Sediment, as a repository of PAHs, plays an important role in environmental information archives^6–9^. Due to the rapid social-economical development and the widespread use of fossil fuels, the deposition flux of PAHs showed an increasing trend year by year^10^. For example, from 2009 to 2016, the concentration range of PAHs in Songhua River increased from 20.50–632.00 ng g^−1^ to 226.70–7086.62 ng g^−1^^11,12^; the values of PAHs in the Persian Gulf had ranged between 72.17 and 277.77 ng g^−1^ in 2009^13^, and increased to 1.98–814.00 ng g^−1^ in 2019^14^.

Taihu Lake, located on the southern edge of the Yangtze River Delta, is the third-largest freshwater lake in China. The Taihu Lake Basin is densely populated, and its economy and industry are also relatively developed^15^. Whether the concentration of PAHs in the Taihu Lake Basin exceeded the standard and whether it posed a health threat to the surrounding population had received widespread attention^16–18^. Since 2000, the concentration of PAHs in the sediments of the Taihu Lake Basin had ranged between 698.00 and 962.00 ng g^−1^, after 209.00–3842.00 ng g^−1^ in 2010, and gradually changed to 4900.00–16,800.00 ng g^−1^ in 2021^19–21^, showing a continuous upward trend. It is imminent to study the concentration, source and carcinogenic risk of polycyclic aromatic hydrocarbons in the sediments of Taihu Lake in the near future. The aims of this study were to (1) quantify 16 PAHs in the sediments of different lake areas in Taihu Lake by gas chromatography-mass spectrometry (GC–MS), (2) explore the occurrence and origins of PAHs in sediments of various-type zones; (3) assess the potential ecological and carcinogenic risks of different PAHs species in Taihu Lake.

## Materials and methods

### Study site and samples collection

**Figure 1:**
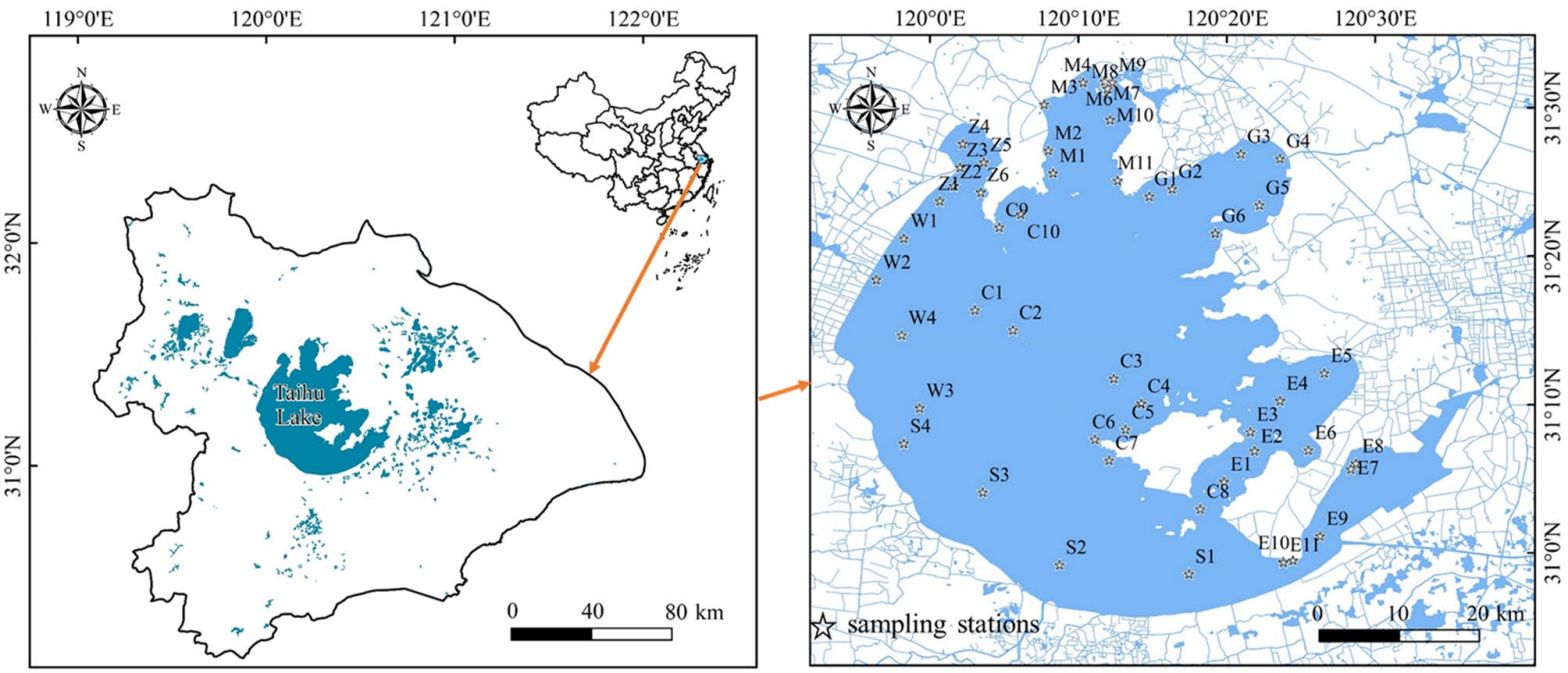
The distributions of specific sampling points. This map was created by ArcGIS 10.2 software and base-map was provided by “Yangtze River Delta Science Data Center, National Earth System Science Data Center, China National Science and Technology Infrastructure” (http://nnu.geodata.cn:8008).

Taihu Lake (30° 55′ 40″–31° 32′ 58″ N, 119° 52′ 32″–120° 36′ 10″ E) located in the lower reaches of Yangtze River, China included Zhushan Bay (ZB), Meiliang Bay (MB), Gonghu Bay (GB), East Taihu area (ETA), South Taihu area (STA), West Taihu area (WTA) and Lake Center (LC). Fifty-two surface sediment samples (Z1–Z6, M1–M11, G1–G6, E1–E11, S1–S4, W1–W4, C1–C10) in above different lake areas of Taihu Lake were sampled through the grab bucket in April 2021 (Fig. 1). The samples were collected into sealed polyethylene bags sterilized by ethylene oxide (Nasco, USA) and placed in an incubator covered with ice packs. The latitudes and longitudes of the sampling locations were obtained through a handheld GPS device (Jiaming, eTrex 221x). After that, the samples were transported to the laboratory and stored in a refrigerator at 4 °C for further analysis.

### Chemicals and reagents

All solvents including dichloromethane and *n*-hexane, were purchased from Leybold (Germany) in HPLC grade. Reagents of 16 PAHs were purchased from AccuStandard (USA) with purity over 98%. The PAHs solutions, which contained all the analytes, was prepared in *n*-hexane and stored at 4 °C in the dark. Water was purified in an Aquapro Ultrapure Water System.

Extraction methods of organic materials. After vacuum freeze-drying and removing shellfish and large plant roots, the samples were ground with an agate mortar and then sieved with a standard 100-mesh sieve (particle size < 0.15 mm).

Approximately (2 ± 0.0001) g of the freeze-dried sample was transferred into a 34-mL extraction cell that had been pre-covered with a gasket and well mixed. Using *n*-hexane/dichloromethane solution (1:1, v:v) as the solvent, the mixed sample was put into the accelerated solvent extractor (ASE) with a 350 system (Thermo Scientific, USA).The extract was transferred to a round bottom flask and concentrated to 2 mL by a vacuum rotary evaporator in a 40℃ water bath. A chromatographic column was successfully prepared by filling 1.5 g of anhydrous sodium sulfate, 1 g of silica gel, and 1.5 g of anhydrous sodium sulfate from top to bottom. The extract was transferred to the prepared chromatographic column and washed with 15 mL of *n*-hexane/dichloromethane mixture (1:1, v:v) and 5 mL of *n*-hexane, and the rinsing fluid was collected to the corresponding flask. The collected liquid was evaporated to 0.5 mL by the rotary evaporator again. The residual collection liquid in the flask was rinsed with *n*-hexane, and the final volume was adjusted to 1 mL^22–24^.

Determination of PAH concentration and composition. In a GC–MS system (Agilent 8860–5977, USA), the pretreated sample was analyzed after passing through a DB-5MS (0.25-μm film thickness, 30 m × 0.25 mm i.d.) silica capillary column. The system used 1 μL splitless injection and 1 mL min^−1^ of high purity (99.999%) helium as the carrier gas. The temperature of the injector was 250 °C while the detector was 280 °C. The system procedure was as follows: hold at 50 °C for 1 min, to 180 °C at 15 °C min^−1^, then to 280 °C at 5 °C min^−1^, and hold for 5 min. The mass detector was maintained at 70 eV and the ion source temperature was 280 °C. 16 PAHs were measured in full scan mode (50–550 amu).

Assessment of PAH pollution level. The ecological risk assessment of PAHs in sediments was frequently carried out using effects range-low (ERL) and effects range-median (ERM)^25,26^. According to the evaluation criteria, when the concentration of PAHs was lower than the value of ERL, there was no adverse effect of PAHs in the area; while the concentration of PAHs was higher than the ERM value, indicating that the regional PAHs were harmful to the biological community^27,28^.

The carcinogenic potential of PAHs was appraised through the equivalent of Benzo(a)pyrene (BaPE)^29^. The value of BaPE was calculated by the concentrations weighted of PAH with the carcinogenic potential of individual PAHs^30^. The calculation formula of BaPE was shown in Eq. (1).

1 $$V_{{B{\text{a}}PE}} = 0.06V_{{B{\text{a}}A}} + 0.07V_{{B{\text{b}}F}} + 0.07V_{{B{\text{k}}F}} + V_{BaP} + 0.6V_{DahA} + 0.08V_{{I{\text{cd}}P}}$$

where *V* represents the value of BaPE; BaA, BbF, BkF, BaP, DahA and IcdP mean Benzo(a)anthracene, Benzo(b)fluoranthene, Benzo(k)fluoranthene, Benzo(a)pyrene, Dibenzo(a,h)anthracene and Indeno(1,2,3-cd)pyrene, respectively.

### Quality control and quality assurance

All glass containers involved in the experiment were baked at 500 ℃ for 4 h in advance. A seven-point calibration curve (50, 100, 250, 500, 1000, 2000, and 2500 ng mL^−1^) with correlation coefficients (R^2^ > 0.996) was selected in combination with an external standard method to quantify PAHs. No target compound was detected in the method blanks. The average recovery rates of PAHs were > 75% in all samples, and the obtained concentration does not pass the recovery rate correction. In repeated samples, the relative standard deviations of PAHs were less than 10%. This study was based on the dry weight of surface sediments samples.

### Statistical analysis

All statistical data were conducted using the statistical procedures (SPSS 21.0, Inc. Chicago, USA). The values of 16 PAHs were statistically analysed, including two rings: Naphthalene (Nap); 3 rings: Acenaphthylene (Acy), Acenaphthene (Ace), Phenanthrene (Phe), Anthracene (Ant) and Fluorene (Flu); 4 rings: Fluoranthene (Flua), Pyrene (Pyr), Benzo(a)anthracene (BaA), and Chrysene (Chry); 5 rings: Benzo(a)pyrene (BaP), Benzo(b)fluoranthene (BbF), Benzo(k)fluoranthene (BkF), Dibenzo(a,h)anthracene (DahA); 6 rings: Indeno(1,2,3-cd)pyrene (IcdP) and Benzo(g,hi)perylene (BghiP). The probable origin of the PAHs, including pyrogenic or petrogenic was determined, and the following isomeric relationships were calculated: Ant/(Ant + Phe), BaA/(BaA + Chry), Flua/(Flua + Pyr), and IcdP/(IcdP + BghiP). Principal component analysis (PCA) was applied to perform dimensionality reduction processing analysis on the original data. The correlation coefficients between the variables were combined into fewer factors, through the correlation between factors to determine the pollution source of PAHs in sediments.

## Results and discussion

### Spatial characteristics of PAH concentration in surface sediments

The total and individual concentrations of PAHs in surface sediments collected from the different locations of Taihu Lake were shown in Table 1. Sixteen kinds of PAH were detected in the surface sediments of Taihu Lake in this study, with concentrations ranging between 1381.48 and 4682.16 ng g^−1^ (average 3032 ng g^−1^). Compared to previous studies, the average PAH concentration of Taihu Lake in 2021 illustrated a significant upward trend during the past 20 years. In 2000, this value was 830 ng g^−1^^19^, and rose to 2026 ng g^−1^ in 2010^20^, which increased by 265% and 50%, respectively. For individual PAH compounds, the values of DahA and IcdP were below the detection limit in most samples, while the concentrations of BghiP in the vast majority of samples were higher. Ace had the lowest contents in each sample. The concentration of ∑_16_PAHs in the ZB lake area was much higher than in the other regions, and the same study results have appeared many times in previous studies^31,32^. The sources of agricultural, industrial, and domestic sewage in the surrounding watershed of ZB lake area were higher than the other locations^33^, resulting in higher PAH concentrations than other areas. The concentration in the northern Taihu Lake was higher than that in the south, and the surrounding concentration was higher than the central region, which mainly depended on the anthropogenic activities, i.e., industrial and agricultural development around the area^34^. The concentrations of pollutants in the central area of the lake were the lowest.

**Table 1:** The concentration of PAHs at different sampling points in Taihu Lake.

Sample points	Nap	Acy	Ace	Flu	Phe	Ant	Flua	Pyr	BaA	Chry	BbF	BkF	BaP	DahA	IcdP	BghiP	∑16PAHs	Average of ∑16PAHs
Rings	2	3	3	3	3	3	4	4	4	4	5	5	5	5	6	6		
	ng g^−1^ (dry weight)																	
**LC**																		
C1	9.01	26.16	7.02	53.60	100.23	120.97	171.92	174.31	271.24	208.48	271.96	313.52	161.60	ND	ND	385.14	2275.17	2125.66 ± 432.32
C2	0.00	39.41	2.73	77.40	138.22	179.25	234.03	242.45	399.80	295.11	390.23	ND	235.24	ND	ND	ND	2233.86	
C3	0.00	38.76	ND	77.66	139.05	177.84	243.72	249.01	402.08	301.25	383.14	ND	237.29	ND	ND	570.52	2820.32	
C4	0.00	27.24	1.45	55.67	106.26	118.00	177.71	174.67	272.19	209.09	273.82	ND	ND	ND	ND	380.90	1797.00	
C5	0.00	26.80	0.68	55.12	106.58	122.71	174.78	173.25	272.95	207.19	ND	ND	154.25	ND	ND	380.64	1674.96	
C6	0.00	38.97	1.61	79.44	145.34	178.66	245.42	247.66	403.89	304.31	387.51	463.70	ND	ND	ND	ND	2496.52	
C7	0.00	25.92	0.00	52.16	93.48	120.74	157.06	160.91	266.33	198.53	ND	306.35	ND	ND	ND	ND	1381.48	
C8	0.00	26.68	0.00	54.67	96.57	158.51	178.52	174.56	272.23	210.04	285.00	309.99	156.51	ND	ND	378.26	2301.53	
C9	0.00	ND	0.03	77.18	138.89	184.07	239.46	248.21	406.00	299.65	ND	ND	233.75	ND	ND	567.37	2394.61	
C10	69.74	28.90	1.37	56.58	103.12	130.86	177.26	176.21	274.86	210.62	267.49	ND	ND	ND	ND	384.20	1881.19	
**ETA**																		
E1	0.00	26.30	6.54	54.13	98.60	187.68	181.79	176.67	276.72	211.70	277.20	309.48	164.10	ND	ND	393.96	2364.86	2540.04 ± 841.59
E2	0.00	25.99	ND	53.58	103.32	176.03	186.92	180.44	276.39	ND	272.36	ND	156.58	ND	ND	381.25	1812.86	
E3	0.05	26.08	ND	53.91	100.17	151.70	188.31	189.15	277.62	198.29	260.77	304.49	175.86	ND	ND	386.80	2313.22	
E4	0.00	26.29	2.18	56.59	107.06	184.51	185.69	179.53	274.74	203.58	270.34	313.25	160.22	ND	ND	378.93	2342.93	
E5	0.00	27.78	0.07	64.55	147.87	218.04	205.36	200.14	289.71	227.57	318.54	310.26	176.81	ND	648.89	413.54	2600.24	
E6	0.00	27.07	ND	55.47	102.11	142.38	170.14	167.73	271.69	191.22	263.80	ND	ND	ND	ND	ND	1391.63	
E7	10.98	32.81	5.17	65.62	140.49	174.12	247.72	239.08	314.24	264.44	345.82	302.68	262.66	549.66	646.07	478.18	4082.56	
E8	0.00	40.86	1.82	85.31	163.16	245.64	266.84	264.57	413.63	306.67	396.91	452.88	259.32	ND	ND	573.25	3470.87	
E9	48.99	31.02	3.14	69.25	175.53	222.23	289.06	255.16	305.16	277.12	307.12	320.41	197.01	ND	ND	436.08	3583.36	
E10	0.00	25.66	ND	52.95	94.61	161.53	160.26	165.29	269.71	199.09	259.89	309.53	ND	ND	ND	ND	1698.52	
E11	0.00	26.38	ND	52.61	93.90	145.41	176.78	173.06	273.78	198.93	281.31	315.80	158.90	ND	ND	382.49	2279.35	
**GB**																		
G1	0.32	26.09	0.00	50.90	94.20	122.31	164.99	167.56	272.23	191.65	262.08	ND	158.39	ND	ND	378.04	1888.75	2329.35 ± 687.44
G2	0.00	25.67	0.00	53.28	117.15	125.16	183.29	181.58	276.82	209.93	270.96	305.16	157.18	ND	ND	384.10	2290.29	
G3	25.74	27.63	3.69	57.67	115.80	128.13	221.14	207.84	288.55	233.93	300.87	314.77	169.85	ND	ND	425.43	2521.02	
G4	11.38	41.99	2.67	81.26	152.52	189.97	299.67	292.49	427.29	334.47	418.40	456.97	238.74	ND	ND	600.29	3548.12	
G5	0.00	25.88	0.86	53.46	106.83	123.81	165.50	166.17	269.50	200.80	270.20	ND	155.49	ND	ND	ND	1538.49	
G6	0.00	38.60	ND	75.82	131.65	178.14	225.79	234.44	399.03	287.63	388.18	ND	230.13	ND	ND	ND	2189.40	
**MB**																		
M1	42.47	ND	2.56	80.96	144.62	182.77	249.23	251.49	406.63	306.66	404.14	444.51	252.18	ND	622.55	570.21	3338.42	2641.63 ± 830.10
M2	71.41	ND	2.30	60.05	121.38	142.02	203.12	195.60	283.51	214.22	ND	319.36	162.39	ND	ND	392.40	2167.76	
M3	69.78	31.20	4.67	64.22	187.56	156.75	365.56	335.70	375.83	324.39	328.47	338.64	257.65	550.53	ND	459.87	4473.36	
M4	21.42	25.88	5.06	56.74	109.32	124.29	159.73	164.31	269.94	199.14	257.21	302.91	153.66	ND	ND	ND	1849.61	
M5	7.27	41.05	0.16	81.07	149.12	184.10	263.18	270.94	416.91	312.56	ND	ND	ND	ND	ND	569.36	2295.72	
M6	0.00	38.53	4.15	78.29	139.17	177.86	240.92	251.80	410.17	299.46	ND	451.71	242.09	ND	ND	ND	2334.15	
M7	49.39	29.59	4.15	64.29	144.29	142.51	236.37	223.03	302.92	242.52	317.38	308.15	183.74	ND	ND	419.88	2668.19	
M8	0.00	38.85	ND	78.67	156.97	186.38	299.80	298.40	437.93	332.15	443.84	468.33	250.84	ND	ND	615.11	3607.27	
M9	0.00	26.85	1.54	57.54	114.45	134.38	204.27	200.42	288.68	226.21	280.05	314.10	165.31	ND	ND	416.82	2430.63	
M10	16.19	26.60	ND	54.53	102.36	127.17	177.27	174.80	274.88	205.07	262.19	ND	ND	ND	ND	384.85	1805.90	
M11	0.00	38.92	8.44	77.20	136.29	181.26	242.95	247.37	406.50	299.80	ND	448.16	ND	ND	ND	ND	2086.90	
**STA**																		
S1	0.00	27.28	3.40	53.30	97.36	163.02	167.68	169.49	270.00	205.80	264.17	306.54	ND	ND	ND	379.59	2107.63	2254.87 ± 356.95
S2	0.00	38.75	0.00	76.53	143.56	177.52	252.47	259.07	409.71	305.27	426.65	ND	ND	ND	ND	591.93	2681.47	
S3	18.90	38.44	2.21	76.53	138.35	176.07	232.58	241.23	399.27	294.42	ND	ND	233.03	ND	621.3	ND	1851.03	
S4	63.37	27.67	5.62	60.96	121.27	124.51	182.80	180.50	273.57	216.96	282.45	304.02	154.90	ND	ND	380.74	2379.35	
**WTA**																		
W1	78.15	ND	6.57	65.42	146.75	141.95	248.17	234.96	305.03	247.55	289.74	326.22	–	ND	ND	449.36	3161.19	2356.08 ± 661.73
W2	0.00	29.14	4.23	62.77	133.21	152.42	209.60	210.30	290.97	224.43	289.14	308.11	199.40	ND	ND	419.14	2532.88	
W3	49.50	27.76	1.95	58.77	110.91	123.05	174.66	168.76	272.97	186.93	285.68	301.34	–	ND	ND	378.61	2140.89	
W4	31.27	25.72	3.20	62.90	114.37	130.26	174.95	177.06	274.52	206.78	ND	ND	–	ND	ND	388.30	1589.34	
**ZB**																		
Z1	63.10	28.98	5.23	63.38	135.29	146.74	198.30	198.06	285.57	220.16	276.24	ND	183.60	ND	701.81	415.71	2220.36	3533.27 ± 898.03
Z2	41.89	42.18	0.00	93.48	184.75	204.60	280.61	289.34	419.03	317.72	412.27	463.39	240.43	ND	ND	611.22	3600.90	
Z3	94.80	32.33	9.95	68.93	182.79	171.18	293.40	286.80	328.45	289.14	283.37	329.59	194.13	551.50	913.86	440.92	4259.07	
Z4	44.42	46.39	6.40	92.44	212.31	237.40	328.17	349.91	439.20	355.34	ND	448.49	298.08	ND	ND	ND	2858.56	
Z5	38.96	30.18	4.00	68.39	143.42	164.95	325.77	322.39	416.24	387.20	503.08	415.11	360.92	ND	ND	587.68	4682.16	
Z6	1.35	41.34	0.00	83.01	159.48	212.66	281.45	285.59	426.33	331.13	406.85	476.68	259.13	ND	ND	613.56	3578.55	

ND expresses the sample concentration below the detection limit; LC, ETA, GB, MB, STA, WTA, and ZB represent the different lake areas i.e., Lake Center, East Taihu area, Gonghu Bay, Meiliang Bay, South Taihu area, West Taihu area, and Zhushan Bay, respectively.

**Figure 2:**
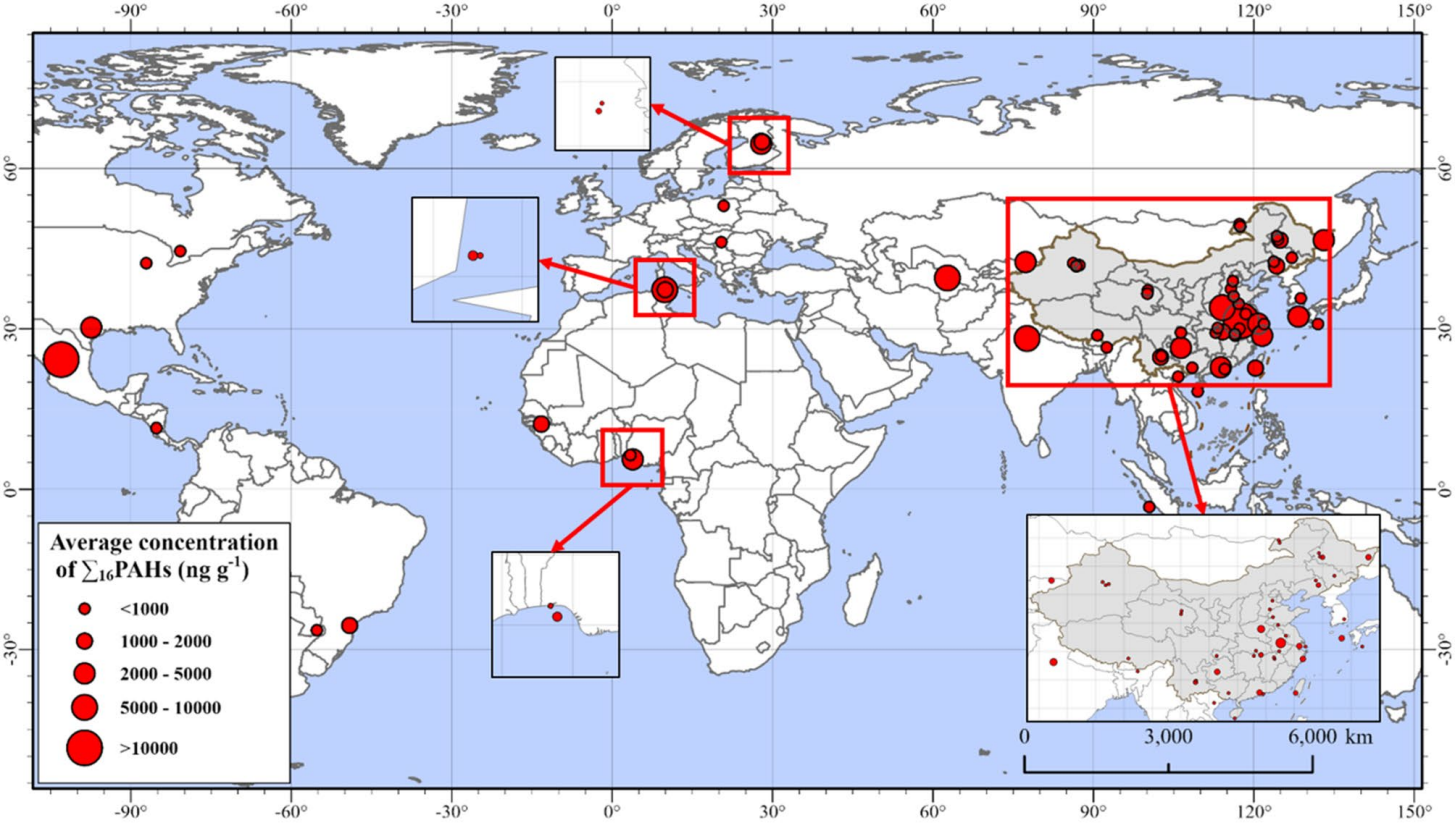
The average concentration of ∑_16_PAHs worldwide (ng g^−1^ dw). All data involved in the figure came from the references in “Supplementary Material” (Table S1); this map was created by ArcGIS 10.2 software and the base-map of the world was provided by ESRI (https://www.esri.com/en-us/home).

The previous worldwide research on the concentration of ∑_16_PAHs in lake sediments had been summarized in this study (Fig. 2). Many studies compared the concentration of ∑_16_PAHs in the surface sediments in China^12,35–37^. Only the average concentrations of ∑_16_PAHs in Chaohu Lake, China^38^ and Tecocomulco Lake, Mexico^39^ were greater than 10,000 ng g^−1^, which could induce aquatic metabolism disorders and promote unpredictable effects on humans’ health. The concentration of ∑_16_PAHs in Taihu Lake was at an upper-middle level in this study.

### Occurrence and compositions of PAHs in different lake areas

**Figure 3:**
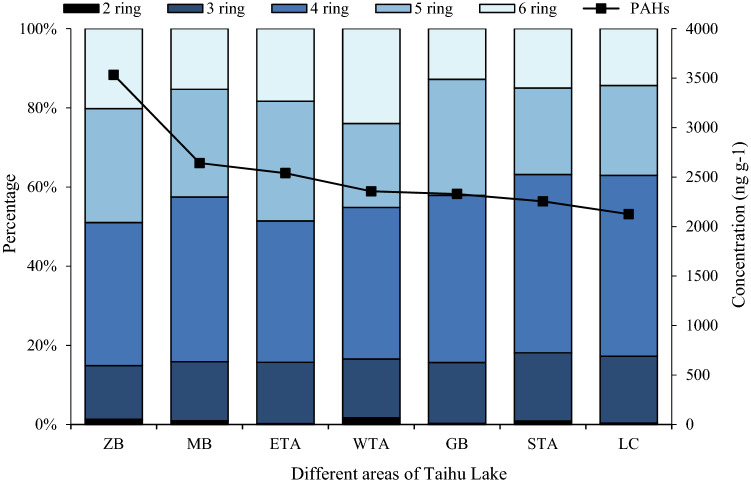
Distribution changes of the PAH monomers in different lake areas of Taihu Lake. ZB, MB, ETA, WTA, GB, STA, and LC represent the Zhushan Bay, Meiliang Bay, East Taihu area, West Taihu area Gonghu Bay, South Taihu area, and Lake Center of Taihu Lake, respectively.

According to the difference in the number of benzene rings, the composition characteristics of PAHs had been studied. As shown in Fig. 3, the 4-ring PAHs were dominant, accounting for 35.69–45.02%. A previous study pointed out that medium-molecular-weight PAHs (4-ring) were predominant in stream sediments in industrial park^40^, indicating that the 4-ring predominance occurred in areas with more serious industrial pollution. But in this study, the top 4 regions with higher industrial pollution (ZB, MB, ETA, WTA) showed high-molecular-weight PAHs (5–6-ring) were dominant. Studies^24,28,41^ have shown that high-temperature combustion of organic materials required for industrial production and vehicle emission will release a higher proportion of 4–6-ring PAHs, while the dominance of 5–6 rings indicates the sources mainly derived from the vehicle emission. The rapid urbanization and traffic jams in these regions resulted in the difference in the occurrence of individual PAHs.The low-molecular-weight (2–3-ring) PAHs were mainly from low-temperature combustion processes and petroleum-derived sources^41^, and medium to high-molecular-weight PAHs were mainly derived from pyrolysis^42^, of which 5 and 6 rings were sources from anthropogenic pyrolysis^7,43^, which preliminarily proved the main source of the study area during the sampling period in Taihu Lake.

### Source appointment of PAHs in the sediments and influencing factors

The sources of PAHs could be identified based on the molecular diagnostic ratio of PAH in the samples^44,45^. The ratio of Ant/(Ant + Phe) greater than 0.10 meant combustion source, less than 0.10 represented oil source; Flt/(Flt + Pyr) between 0.40 and 0.50 represented the burning particles from liquid fossil fuels (vehicles and crude oil), greater than 0.50, mainly from grass, wood, and coal combustion; the ratio of BaA/(BaA + Chry) greater than 0.35 indicated combustion was the main source, and less than 0.20 was the source of petroleum; IcdP/(IcdP + BghiP) between 0.20 and 0.50 meant burning of liquid fossil fuels, values greater than 0.50 indicated grass, wood, and coal combustion.

**Figure 4:**
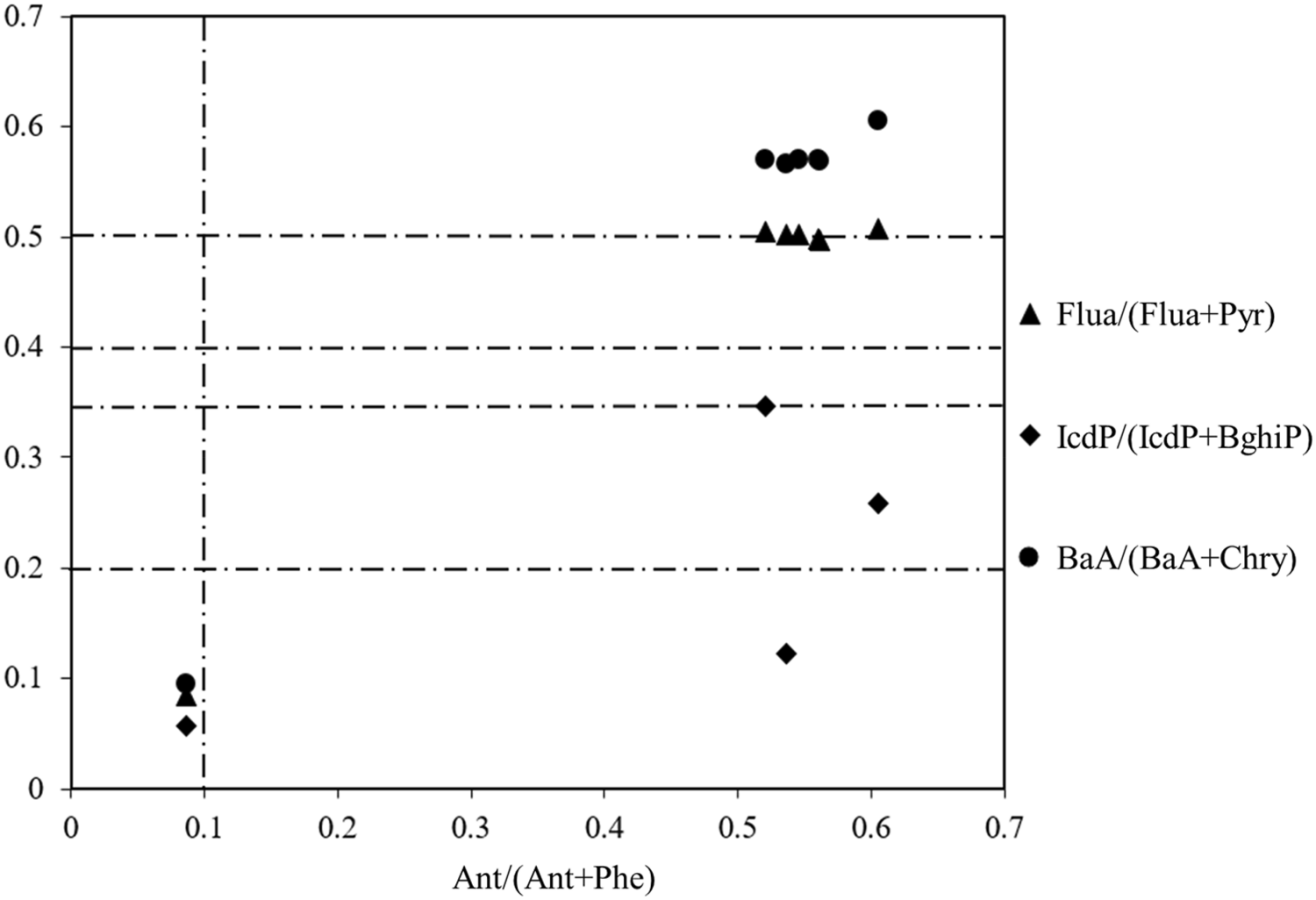
Source apportionment of PAHs in the sediments of Taihu Lake.

The proportion of PAH monomers in different regions was calculated in this study (Fig. 4). The ratios of Ant/(Ant + Phe) in most sediment samples from Taihu Lake were greater than 0.10, while in ZB was less than 0.10. The same phenomenon appeared in the BaA/(BaA + Chry) ratios, in addition to ZB, ratios were greater than 0.35, indicating that except for ZB, where was the petroleum source, the main sources of PAHs in other regions from Taihu Lake were combustion. Zhushan Bay was one of the most polluted areas in Taihu Lake, with developed industries and huge petroleum consumption, which was an important source of PAHs^46^. The values of Flua/(Flua + Pyr) were mainly greater than 0.50, and the values in the lake areas of LC and STA were between 0.40 and 0.50, which represented the PAHs in LC and STA may be derived from liquid fossil fuels (vehicles and crude oil). The IcdP/(IcdP + BghiP) values in the lake areas of ETA and WTA ranged between 0.20 and 0.50, which meant the burning of liquid fossil fuels^44,47^. Therefore, it was further derived the PAHs in ZB mainly from the petroleum source, and the main sources of GB and MB were the burning of grass, wood, and coal. While PAHs in other areas of Taihu Lake were from the combustion of liquid fossil fuels, which was consistent with the previous studies^48,49^.

**Figure 5:**
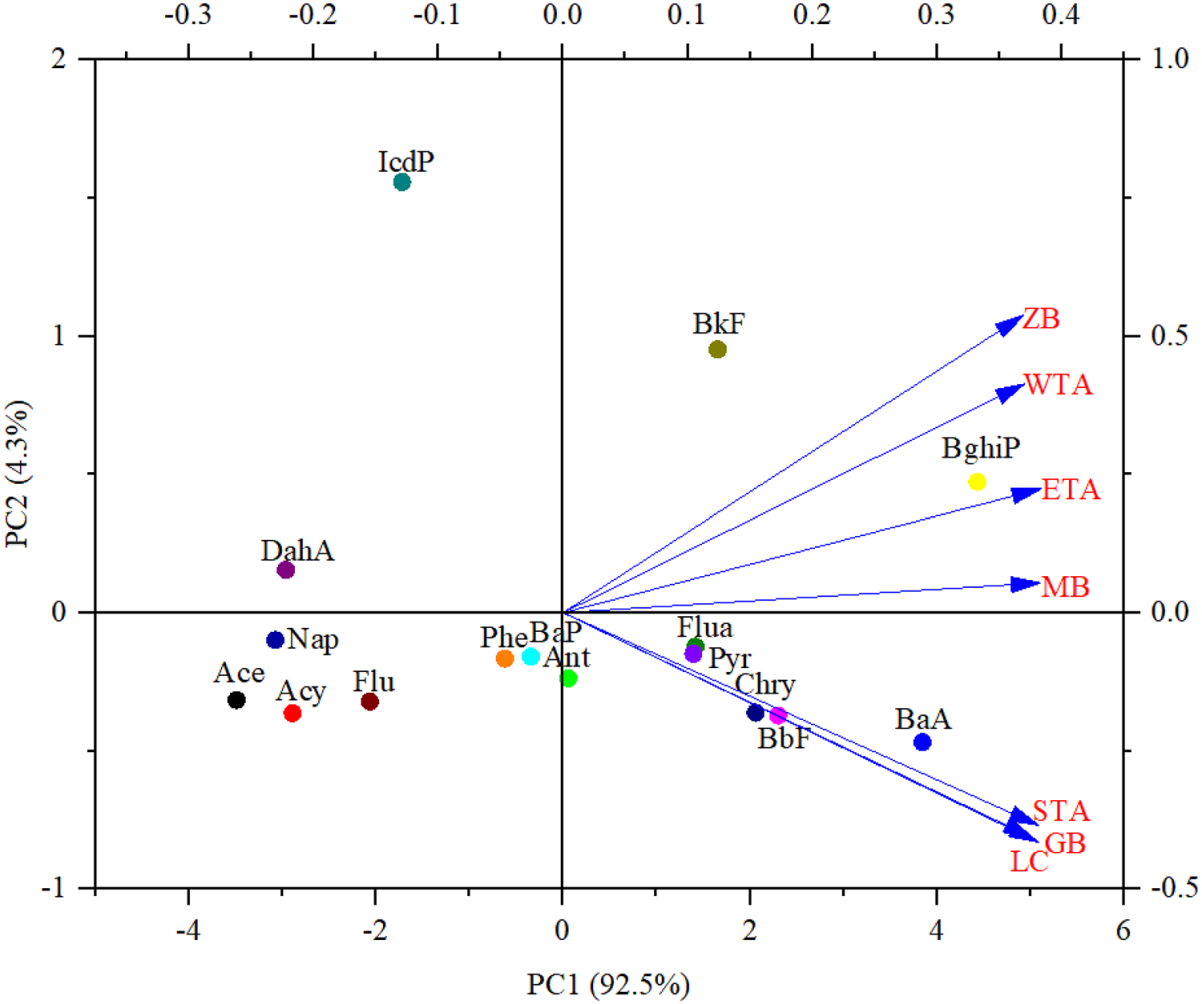
PCA determination of PAH sources in the sediments of Taihu Lake.

To further determine the source of PAHs in the entire Taihu Lake area, PCA was selected as an effective identification tool. Figure 5 illustrated the score plot of the first two components (PC) of PAHs in surface sediments of Taihu Lake, accounting for 96.80% of the variance. PC_1_ was responsible for 92.50% of the total variance and exhibited high loading for BghiP, BaA, BbF, Chry, BkF, Flua, and Pyr (4.43, 3.85, 2.30, 2.06, 1.66, 1.41, and 1.40). PC_2_ was responsible for 4.30% of the total variance, and mostly due to IcdP, BghiP, and DahA (1.56, 0.47, and 0.15). It has been proved in previous studies, BghiP, BkF, and IcdP were derived from vehicle exhaust, BaA and Chry were the products of petroleum combustion, BbF was from the high-temperature combustion, and Flua and Pyr came from coal-burning^50–52^. To further derive, the main sources of PAHs in surface sediments from Taihu Lake were man-made sources, including vehicle exhaust, petroleum combustion, and high-temperature combustion, which was consistent with the results of the PAH molecular diagnostic ratio.

### Risk assessment and ecological suggestions

The biological toxicity assessment of PAHs in different sediments from Taihu Lake was compared. It was shown (Table 2) that the concentrations of Nap, Ace, Phe, Flua, Pyr, and BaP in all sampling sites were lower than the ERL values, which would not cause adverse effects on the environment. The concentrations of Chry, BkF, BbF, and BghiP at some sampling points were higher than the ERL value but lower than the ERM value, which had little impact on the environment. It was worth noting that the DahA concentration was much higher than the ERM value, which might greatly influence the ecological community. And the IcdP biological toxicity was relatively high, which needs to be monitored.

**Table 2:** The biological toxicity assessment form of PAHs in different sediments of Taihu Lake (ng g^−1^ dw).

PAHs	ERL	ERM	Average concentration in different lake areas							Concentration range
			ZB	MB	GB	ETA	STA	WTA	LC	
Nap	160.00	2100.00	47.42	25.27	6.24	5.46	20.57	39.73	7.87	0.00–94.80
Acy	16.00	500.00	36.90	27.04	30.98	28.75	33.04	20.66	27.88	NA–46.39
Ace	44.00	640.00	4.26	3.00	1.20	1.72	2.80	3.99	1.49	NA–9.95
Flu	19.00	540.00	78.27	68.51	62.07	60.36	66.83	62.47	63.95	50.90–93.48
Phe	240.00	1500.00	169.67	136.87	119.69	120.62	125.14	126.31	116.77	93.48–212.31
Ant	85.30	1100.00	189.59	158.14	144.59	182.66	160.28	136.92	149.16	118.00–245.64
Flua	600.00	5100.00	284.62	240.21	210.06	205.35	208.88	201.85	199.99	157.06–365.56
Pyr	665.00	2500.00	288.68	237.62	208.35	199.17	212.57	197.99	202.12	160.91–349.91
BaA	261.00	1600.00	385.80	352.17	322.24	294.85	338.14	285.87	324.16	266.33–439.20
Chry	384.00	2800.00	316.78	269.29	243.06	207.15	255.61	216.42	244.43	NA–387.20
BkF	280.00	1620.00	355.54	308.72	179.48	267.16	152.64	233.92	139.36	NA–503.08
BbF	320.00	1880.00	313.63	208.48	318.45	295.82	243.31	216.14	225.91	NA–476.68
BaP	430.00	1600.00	256.05	151.62	184.96	155.59	96.98	49.85	117.86	NA–360.92
DahA	63.40	260.00	91.92	50.05	NA	19.97	NA	NA	NA	NA–551.50
IcdP	–	–	269.28	56.60	NA	117.72	NA	155.32	NA	NA–913.86
BghiP	430.00	1600.00	444.85	348.05	297.97	347.68	338.07	408.85	304.70	NA–615.11

ZB, MB, ETA, WTA, GB, STA, and LC represent the Zhushan Bay, Meiliang Bay, East Taihu area, West Taihu area Gonghu Bay, South Taihu area, and Lake Center, respectively.

Due to the high carcinogenicity of DahA and IcdP^53,54^, the carcinogenic risk of DahA and IcdP had been further studied in this study. The BaPE was a useful indicator for quantitatively assessing the potential carcinogenic risks of PAHs^55,56^.

**Figure 6:**
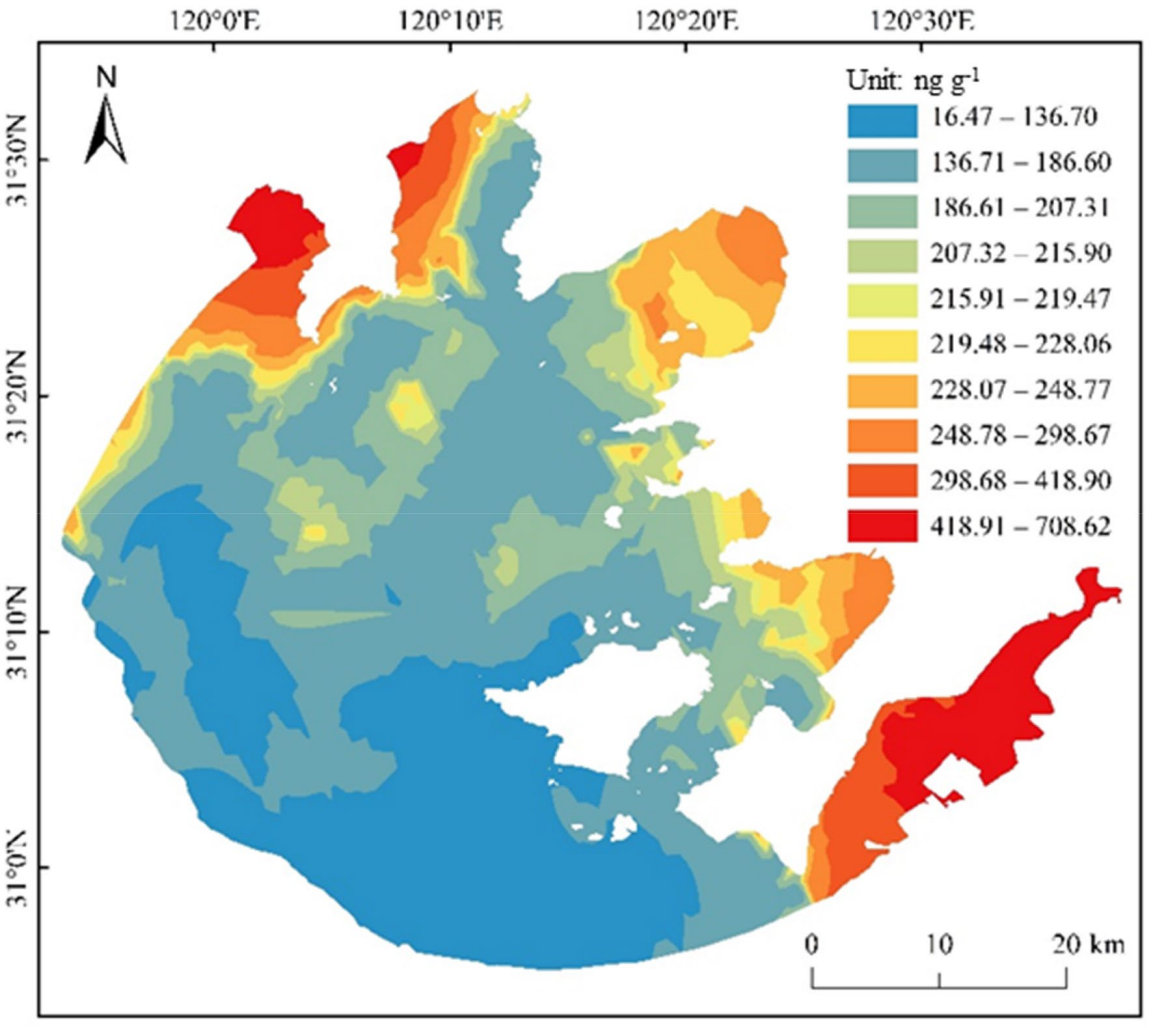
The spatial distribution of BaPE in sediments of Taihu Lake. This map was created by ArcGIS 10.2 software and base-map was provided by “Yangtze River Delta Science Data Center, National Earth System Science Data Center, China National Science and Technology Infrastructure”, (http://nnu.geodata.cn:8008).

The BaPE value in this study was calculated through the data of Table 1, and the results were shown in Fig. 6. It could be seen from Fig. 6 that the BaPE values for 52 surface sediments samples of Taihu Lake varied from 16.47 to 708.62 ng g^−1^ dw. The high BaPE levels were noticed at sites E7, M3, Z3, and Z5 indicating the relatively high toxicity of PAHs in these sites compared to other sites. The PAHs in ZB, MB, and the southeast area of Taihu Lake had higher carcinogenic risks. Compared with 2012, the risk of PAHs had increased obviously^33^. PAHs are derived from both natural and anthropogenic activities. With the enhanced anthropogenic stressors, the PAHs discharge increased accompanied by higher high-ring monomers. Terrestrial runoff, atmospheric deposition, and internal sources are the key PAH sources in lakes. Four suggestions on reducing organic pollutants and ecological restoration for similar lakes were proposed: (1) adjustment of energy structure and industrial structure, (2) construction of lake buffer to decrease the organic pollutant input, (3) in-situ degradation of PAHs in different lake areas, (4) dredging of lake sediments regularly to ease the burden of endogenous release on the water environment.

## Conclusions

In this work, 16 priority PAHs in spatial sediments from Taihu Lake, China were compared, and the possible source contributions were estimated. The current pollution levels of PAHs in different lake areas were at a medium-to-high global level. The distributions of PAHs around the basin were higher than those in the central areas of the lake indicating the high frequency of anthropogenic activities, high economic and development levels in the lake area. The high population density in the study area presented a more comprehensive view to evaluate the regional impact on PAH sources. PAHs in Zhushan Bay were mainly sourced from petroleum, however, the main origins of PAHs in both Gonghu Bay and Meiliang Bay were derived from the burning of grass, wood, and coal. For other lake areas, the combustion of liquid fossil fuels contributed greatly. The ERL and ERM values combining the BaPE values with the difference of lake areas were calculated to evaluate regional impact more comprehensively, which was different from previous results that grass-type lake zones may not necessarily present high regional impacts. PAHs in Zhushan Bay, Meiliang Bay, and the southeast of Taihu Lake had a high risk of carcinogenesis and showed an upward trend year by year.

## Supplementary Information

Supplementary Information.
