# Supplementary material for: Spatial occurrence and sources of PAHs in sediments drive the ecological and health risk of Taihu Lake in China
Source: Sci Rep. 2022 Mar 7;12:3668. doi: 10.1038/s41598-022-07507-7 (PMC8901641; doi:10.1038/s41598-022-07507-7)
Supplement: Supplementary file 1 — Supplementary Information. [file 41598_2022_7507_MOESM1_ESM.docx]

**Supplementary materials**

**Table S1 The specific data of the relevant study involved in Fig. 2**

| **Lake Name** | **Located** | **Longitude** | **Latitude** | **Minimum concentration**  **(ng g^-1^)** | **Maximum concentration**  **(ng g^-1^)** | **Average concentration**  **(ng g^-1^)** | **References** |
| --- | --- | --- | --- | --- | --- | --- | --- |
| Nakdong River | Korea | 128.77 | 35.7 | 0 | 969.3 | 484.65 | 1 |
| Yamzho Yumco Lake | China | 90.77 | 28.75 | 0.91 | 57.97 | 29.44 | 2 |
| Lake Hongze | China | 118.5 | 32.79 | 299 | 723 | 511 | 3 |
| Bosten Lake | China | 86.82 | 41.73 | 57.37 | 360.24 | 208.805 | 4 |
| Bosten Lake | China | 86.77 | 41.69 | 51.07 | 583.73 | 317.4 | 5 |
| Poyang Lake | China | 116.5 | 28.68 | 16 .4 | 775 | 387.5 | 6 |
| La Salada shallow lake | Argentina | 62.7 | 39.45 | 12.55 | 10679.48 | 5346.015 | 7 |
| Yangtze River | China | 114.18 | 29.44 | 572.7 | 1766.2 | 1169.45 | 8 |
| Honghu Lake | China | 113.22 | 30.11 | 93 | 431 | 262 | 9 |
| Chagan Lake | China | 117.29 | 49.59 | 305.9 | 1214.42 | 760.16 | 10 |
| Lake Qinghai | China | 100.06 | 36.58 | 30.4 | 125.2 | 77.8 | 11 |
| Lake Vesijarvi | China | 27.88 | 64.63 | 100 | 8500 | 4300 | 12 |
| Yangtze River | China | 121.55 | 28.7 | 15.14 | 5355 | 2685.07 | 13 |
| Songhua Lake | China | 127.1 | 43.29 | 23.1 | 554.8 | 288.95 | 14 |
| Barigui River | Brazil | -49.09 | -25.48 | 39 | 2350 | 1194.5 | 15 |
| Itaipu Lake | Brazil | -55.25 | -26.36 | 35.21 | 685.37 | 360.29 | 16 |
| Hulun La | China | 117.47 | 49.11 | 282.9 | 1056.9 | 669.9 | 17 |
| Baiyangdian Lake | China | 116.11 | 38.93 | 23.79 | 329.4 | 176.595 | 18 |
| Weishan Lake | China | 117.16 | 34.69 | 324.93 | 1576.65 | 950.79 | 19 |
| Dishui Lake | China | 121.94 | 30.87 | 90 | 1410 | 750 | 20 |
|  |  | 115.68 | 37.41 | 461.1 | 1497.5 | 979.3 |  |
|  |  | 124.25 | 41.71 | 102.3 | 2240.5 | 1171.4 |  |
| Lake Chaohu | China | 117.61 | 31.54 | 80.82 | 30365.01 | 15222.92 | 21 |
| Lake Hongfeng | China | 106.4 | 26.43 | 2936.1 | 5282.3 | 4109.2 | 22 |
| Hengshui Lake | China | 106.38 | 29.24 | 337.3 | 1604.1 | 970.7 | 23 |
| Lake Michigan | America | -87.12 | 42.24 | 213 | 1291 | 752 | 24 |
| Dongping Lake | China | 116.2 | 36.03 | 77.6 | 628 | 352.8 | 25 |
| Brahmaputra Rivers | India | 92.46 | 26.51 | - | - | 370 | 26 |
| Dianchi Lake | China | 102.6 | 24.63 | 746 | 2293 | 1519.5 | 27 |
| Kaidu River | China | 86.22 | 42.24 | 20.8 | 491 | 255.9 | 28 |
| Bosten Lake | China | 87.36 | 41.87 | 25.2 | 491 | 258.1 | 29 |
| Dianshan Lake | China | 120.85 | 30.97 | 2805 | 4130 | 3467.5 | 30 |
| Ichkeul and Bizerte Lagoon | Tunisia | 9.87 | 37.27 | 122 | 19600 | 9861 | 31 |
| Taihu Lake | China | 112.88 | 29.27 | 206.56 | 1058.98 | 632.77 | 32 |
| Dishui Lake | China | 131.98 | 30.89 | 74.03 | 579.2 | 326.615 | 33 |
| Lake of rural oil field area, industrial urban oil field area, and urban industrialized area | China | 124.93 | 46.59 | 293 | 2247 | 1270 | 34 |
|  |  | 124.56 | 46.69 | 215 | 1308 | 761.5 |  |
|  |  | 124.31 | 47.33 | 140 | 363 | 251.5 |  |
| Lake Issyk-Kul and Lake Son-Kul | Kyrgyzstan | 77.27 | 42.51 | 52 | 9439 | 4745.5 | 35 |
| Lake Nicaragua | Nicaragua | -85.21 | 11.45 | 10 | 640 | 325 | 36 |
| Dianchi Lake | China | 102.71 | 24.88 | 92.31 | 1546.78 | 819.545 | 37 |
| River located in the state of Bahia | Brazil | -13.22 | 12.18 | 260 | 2100 | 1180 | 38 |
| Lake Poyang | China | 116.41 | 29 | 41.3 | 384 | 212.65 | 39 |
| Bizerte Lagoon/Ichkeul Lake | Tunisia | 9.877 | 37.27 | 124 | 3371 | 1747.5 | 40 |
| Lady Bird Lake | America | -97.44 | 30.2 | - | - | 4500 | 41 |
| Jaimsainves Lake | Finland | 28.04 | 65 | 407 | 3294 | 1850.5 | 42 |
| Liaohe River | China | 123.75 | 42.55 | 256.8 | 560.1 | 408.45 | 43 |
| Songgang River | China | 113.84 | 22.77 | 208.7 | 7709.8 | 3959.25 | 44 |
| Lake Tecocomulco | Mexico | -103.02 | 24.21 | - | - | 10927 | 45 |
| Luda Lake | Serbia | 20.39 | 46.22 | 50 | 125 | 87.5 | 46 |
| Lake Bangong Co | China | 78.69 | 34.29 | 21.5 | 43.1 | 32.3 | 47 |
| Laurentian Great Lakes | Canada | -80.75 | 44.55 | 21.5 | 43.1 | 32.3 | 48 |
| Qinghai Lake | China | 100.14 | 37.18 | 0.03 | 23 | 11.515 | 49 |
| Qingshuiquan | China | 108.47 | 22.72 | 169 | 638.94 | 403.97 | 50 |
| Salt River | China | 120.28 | 22.64 | 257.7 | 609.5 | 433.6 | 51 |
| Yamuna River | India | 77.6 | 28.17 | 343 | 2940 | 1641.5 | 52 |
| Sanya River | China | 109.48 | 18.24 | 1576 | 12546 | 7061 | 53 |
| Huai River | China | 117.35 | 30.02 | 3.23 | 493 | 248.115 | 54 |
| Songhua River | China | 133.1 | 46.59 | 533.15 | 1422.83 | 977.99 | 55 |
| Shaying River | China | 114.12 | 33.93 | 226.7 | 7086.62 | 3656.66 | 56 |
| Lagos lagoon Lake | Africa | 3.42 | 6.4 | 64.27 | 11433.63 | 5748.95 | 57 |
| River in Ulsan | Korea | 128.36 | 32.29 | 302 | 1290 | 796 | 58 |
| Liuxi River | China | 114.52 | 22.47 | 207 | 5306 | 2756.5 | 59 |
| Lagos lagoon Lake | Africa | 3.86 | 5.61 | 296.26 | 888.14 | 592.2 | 60 |
| KimNguu river | Vietnam | 105.86 | 20.99 | 1430 | 5900 | 3665 | 61 |
| Panyang River | China | 97.04 | -6.57 | 4.73 | 36.34 | 20.535 | 62 |
| Curonian Lagoon and the Nemunas River Delta | Baltic Sea | 20.88 | 53 | 12.91 | 37.8 | 25.355 | 63 |

**References**

1. Huh, I. A. *et al*. Assessment of Distribution Patterns and Sources for PAHs, OCPs, and Co-PCBs in the Surface Sediments from the Nakdong River Basin, Korea. *Daehan Hwangyeong Gonghag Hoeji* **32**, 656-664. (2010)
2. Wang, Q. *et al*. The sedimentary record of polycyclic aromatic hydrocarbons in Yamzho Yumco Lake: evolution of local sources and adsorption dynamic in the Tibetan Plateau. *Environ Sci Pollut Res* **26**, 18674-18686. <https://doi.org/10.1007/s11356-019-05182-2> (2019).
3. Wu, Z. F. & Tao, Y. Q. Occurrence, sources, bioaccumulation, and air-water exchange fluxes of polycyclic aromatic hydrocarbons in Lake Hongze, China. *J Soils Sediments* **21**, 2969-2980. <https://doi.org/10.1007/s11368-021-02982-3> (2021).
4. Song S. J. *et al*. Spatial and temporal distribution,sources,and ecological risk assessment of polycyclic aromatic hydrocarbons in sediments of the Bosten Lake watershed. *Huanjing Kexue Xuebao* **39**, 2780-2790. (2019) (In Chinese).
5. Sun, H. J., Sun, L. & Zang, S. Y. History of atmospheric PAHs sedimentation and response to human activities in Bosten Lake in Western China. *Arab J Geosci* **14**, 1047. <https://doi.org/10.1007/s12517-021-07432-5> (2021).
6. Zhang, Y. C. *et al*. Spatial-temporal variations and transport process of polycyclic aromatic hydrocarbons in Poyang Lake: Implication for dry-wet cycle impacts. *J Geochem Explor* **226**, 106738. <https://doi.org/10.1016/j.gexplo.2021.106738> (2021).
7. Oliva, A. L. et al. First records of polycyclic aromatic hydrocarbons and metals in sediments from a shallow lake in the Pampean-Patagonian region (Argentina). *Mar Freshw Res* **70**, 1378-1388. <https://doi.org/10.1071/MF18310> (2019).
8. Wan H. B. *et al*. Distribution,source characteristics and ecological risk assessment of polycyclic aromatic hydrocarbons in surface sediments of lakes along the middle reaches of the Yangtze River. *Hu Po Ke Xue* **32**, 1632-1645. (2020) (In Chinese).
9. Zheng, H. *et al*. Source apportionment of polycyclic aromatic carbons (PAHs) in sediment core from Honghu Lake, central China: comparison study of three receptor models. *Environ Sci Pollut Res* **24**, 25899-25911. <https://doi.org/10.1007/s11356-017-0185-x> (2017).
10. Sun H. J., Zang S. Y., Zhang K. & Sun L. Effects of human activities on sedimentary accumulation of PAHs in Chagan Lake. *Huanjing Kexue Xuebao* **40**, 3350-3360. (2020) (In Chinese).
11. Cao, Y. X. *et al*. Distribution, source, and ecological risks of polycyclic aromatic hydrocarbons in Lake Qinghai, China. *Environ Pollut* **266**, 115401. <https://doi.org/10.1016/j.envpol.2020.115401> (2020).
12. Honkonen, O & Rantalainen, A. L. Impact of urbanization on the concentrations and distribution of organic contaminants in boreal lake sediments. *Environ Monit Assess* **185**, 1437-1449. <https://doi.org/10.1007/s10661-012-2643-8> (2013).
13. Jia, T. Q. *et al*. Spatial distribution of polycyclic aromatic hydrocarbons in the water-sediment system near chemical industry parks in the Yangtze River Delta, China. *Sci Total Environ* **754**, 142176. <https://doi.org/10.1016/j.scitotenv.2020.142176> (2021).
14. Cui Z. D. *et al*. Pollution characteristics of polycyclic aromatic hydrocarbons and phthalate esters in surface sediments of in Songhua Lake. *Res Environ Sci* **32**, 15131-1539. (2019) (In Chinese).
15. Machado, K. S. *et al*. Sedimentary record of PAHs in the Barigui River and its relation to the socioeconomic development of Curitiba, Brazil. *Sci Total Environ* **482**, 42-52. <https://doi.org/10.1016/j.scitotenv.2014.02.106> (2014).
16. Kalwa, M., Quinaia, S. P., Pletsch, A. L., Torres, Y. R. & Finger, D. Polycyclic aromatic hydrocarbons in surface sediments of Binacional Itaipu Lake, Brazil: characteristics, sources and toxicity evaluation. *Environ Earth Sci* **72**, 4473-4481. <https://doi.org/10.1007/s12665-014-3347-x> (2014).
17. Sun, H. J., Sun, L. & Zang, S. Y. Variation characteristics and environmental risk assessment of PAHs in sediments of Hulun La in Northern China. *Fresenius Environ Bull* **29**, 8410-8420. (2020).
18. Wang, T. F. *et al*. Distribution characteristics and risk assessment of antibiotics and polycyclic aromatic hydrocarbons in the sediments of desilting demonstration area in Baiyangdian Lake. *Huan jing ke xue* **42**, 5303-5311. (2021) (In Chinese).
19. Zheng X. & Han B. P. Content analysis and assessment of polycyclic aromatic hydrocarbons in surface sediments from Weishan Lake, China. *Nong Ye Huan Jing Ke Xue Xue Bao* **29**, 2185-2191. (2010) (In Chinese).
20. Han R. L. Y. *et al*. Distribution and health risk assessment of polycyclic aromatic hydrocarbons in surface water, sediments and organisms of Dishui Lake and its surrounding water system. *Sheng tai du li xue bao* **15**, 240-251. (2020) (In Chinese).
21. Li, C. C. *et al*. Spatial distribution, potential risk assessment, and source apportionment of polycyclic aromatic hydrocarbons (PAHs) in sediments of Lake Chaohu, China. *Environ Sci Pollut Res* **21**, 12028-12039. <https://doi.org/10.1007/s11356-014-3137-8> (2014).
22. Guo, J. Y. *et al*. Screening level of PAHs in sediment core from Lake Hongfeng, Southwest China. *Arch Environ Contam Toxicol* **60**, 590-596. <https://doi.org/10.1007/s00244-010-9568-4> (2011).
23. Wu, P. *et al*. PAHs pollution characteristics and source analysis of typical lake and reservoir sediments in Jin-Ji-Liao Area. *Huan jing ke xue* **42**, 1791-1800. <https://doi.org/10.13227/j.hjkx.202008046> (2021). (In Chinese).
24. Huang, L., Chernyak, S. M. & Batterman, S. A. PAHs (polycyclic aromatic hydrocarbons), nitro-PAHs, and hopane and sterane biomarkers in sediments of Southern Lake Michigan, USA. *Sci Total Environ* **487**, 173-186. <https://doi.org/10.1016/j.scitotenv.2014.03.131> (2014).
25. Guo, W. *et al*. A 110 year sediment record of polycyclic aromatic hydrocarbons related to economic development and energy consumption in Dongping Lake, North China. *Molecules (Basel, Switzerland)* **26**, 1-13. <https://doi.org/10.3390/molecules26226828> (2021).
26. Khuman, S. N., Chakraborty, P., Cincinelli, A., Snow, D. & Kumar, B. Polycyclic aromatic hydrocarbons in surface waters and riverine sediments of the Hooghly and Brahmaputra Rivers in the Eastern and Northeastern India. *Sci Total Environ* **636**, 751-760. <https://doi.org/10.1016/j.scitotenv.2018.04.109> (2018).
27. Ma, X. H. *et al*. Sediment record of polycyclic aromatic hydrocarbons in Dianchi lake, southwest China: Influence of energy structure changes and economic development. *Chemosphere* **248**, 126015. <https://doi.org/10.1016/j.chemosphere.2020.126015> (2020).
28. Shen, B. B., Wu, J. L., Zhao, Z. H. & Ma, L. Residue and distribution characteristics of organochlorine pesticides and polycyclic aromatic hydrocarbons in different environmental components of the Kaidu River Catchment, Xinjiang. *Bull Mineral Petrol Geochem* **35**, 646-652. (2016) (In Chinese).
29. Shen, B. B., Wu, J. L. & Zhao, Z. H. Organochlorine pesticides and polycyclic aromatic hydrocarbons in water and sediment of the Bosten Lake, Northwest China. *J Arid Land* **9**, 287-298. <https://doi.org/10.1007/s40333-017-0008-4> (2017).
30. Yang, J. *et al*. Occurrence, source, and partition of PAHs, PCBs, and OCPs in the multiphase system of an urban lake, Shanghai. *Appl Geochem* **106**, 17-25. <https://doi.org/10.1016/j.apgeochem.2019.04.023> (2019).
31. Ben Salem, F., Ben Said, O., Mahmoudi, E., Duran, R. & Monperrus, M. Distribution of organic contamination of sediments from Ichkeul Lake and Bizerte Lagoon, Tunisia. *Mar Pollut Bull* **123**, 329-338. <https://doi.org/10.1016/j.marpolbul.2017.09.024> (2017).
32. Zhang, Y. *et al*. Potential source contributions and risk assessment of PAHs in sediments from Taihu Lake, China: Comparison of three receptor models. *Water Res* **46**, 3065-3073. <https://doi.org/10.1016/j.watres.2012.03.006> (2012).
33. Mei, W. P., Ruan, H. H., Wu, H., Le, H. T. & Jiang, M. Distribution and ecological risk assessment of polycyclic aromatic hydrocarbons in sediments from Dishui Lake water system. *Zhongguo huan jing ke xue* **33**, 2069-2074. (2013) (In Chinese).
34. Sun, L., Zang, S. Y. & Sun, H. J. Sources and history of PAHs in lake sediments from oil-producing and industrial areas, northeast China. *Int J Environ Sci Technol* **11**, 2051-2060. <https://doi.org/10.1007/s13762-013-0396-8> (2014).
35. Li, Q. Y., Wu, J. L., Zhou, J. C., Sakiev, K. & Hofmann, D. Occurrence of polycyclic aromatic hydrocarbon (PAH) in soils around two typical lakes in the western Tian Shan Mountains (Kyrgyzstan, Central Asia): Local burden or global distillation? *Ecol Indic* **108**, 105749. <https://doi.org/10.1016/j.ecolind.2019.105749> (2020).
36. Scheibye, K. *et al*. Sediment baseline study of levels and sources of polycyclic aromatic hydrocarbons and heavy metals in Lake Nicaragua. *Chemosphere* **95**, 556-565. <https://doi.org/10.1016/j.chemosphere.2013.09.115> (2014).
37. Hu, X. Y. *et al*. Distribution of 16 polycyclic aromatic hydrocarbons in Dianchi Lake surface sediments after the integrated water environment control project. *Huan jing ke xue* **40**, 3501-3508. <https://doi.org/10.13227/j.hjkx.201901113> (2019). (In Chinese).
38. dos Santos, I. F., Ferreira, S. L. C., Dominguez, C. & Bayona, J. M. Analytical strategies for determining the sources and ecotoxicological risk of PAHs in river sediment. *Microchemical Journal* **137**, 90-97. <https://doi.org/10.1016/j.microc.2017.09.025> (2018).
39. Li Q. Y., Zhao Z. H., Jiang Y. & Zhang L. Vertical distribution characteristics of organochlorine pesticides and polycyclic aromatic hydrocarbons in a sedimentary core from Zhouxi Bay, Lake Poyang. *Hu Po Ke Xue* **28**, 765-774. (2016) (In Chinese).
40. Ben Salem, F., Ben Said, O., Duran, R. & Monperrus, M. Validation of an adapted QuEChERS method for the simultaneous analysis of polycyclic aromatic hydrocarbons, polychlorinated biphenyls and organochlorine pesticides in sediment by gas chromatography-mass spectrometry. *Bull Environ Contam Toxicol* **96**, 678-684. <https://doi.org/10.1007/s00128-016-1770-2> (2016).
41. Van Metre, P. C. & Mahler, B. J. PAH Concentrations in Lake Sediment Decline Following Ban on Coal-Tar-Based Pavement Sealants in Austin, Texas. *Environ Sci Technol* **48**, 7222-7228. <https://doi.org/10.1021/es405691q> (2014).
42. Hyotylainen, T. & Oikari, A. The toxicity and concentrations of PAHs in creosote-contaminated lake sediment. *Chemosphere* **38**, 1135-44. <https://doi.org/10.1016/S0045-6535(98)00362-2> (1999).
43. Zhang, Y. *et al*. Evaluation of AhR-agonists and AhR-agonist activity in sediments of Liaohe River protected areas, China. *Mar Pollut Bull* **115**, 292-296. <https://doi.org/10.1016/j.marpolbul.2016.10.064> (2017).
44. Wang, F. *et al*. Spatial and vertical distribution, composition profiles, sources, and ecological risk assessment of polycyclic aromatic hydrocarbon residues in the sediments of an urban tributary: A case study of the Songgang River, Shenzhen, China. *Environ Pollut* **266**, 115360. <https://doi.org/10.1016/j.envpol.2020.115360> (2020).
45. Romo-Gomez, C., Monks, S., Pulido-Flores, G. & Gordillo-Martinez, A. J. Determination of polycyclic aromatic hydrocarbons (PAHs) in superficial water and sediment of Lake Tecocomulco, Mexico. *Interciencia* **35**, 905-911. (2010).
46. Grba, N. *et al*. Organic and inorganic priority substances in sediments of Luda Lake, a cross-border natural resource on the Ramsar list. *Environ Sci Pollut Res* **24**, 1938-1952. <https://doi.org/10.1007/s11356-016-7904-6> (2017).
47. Lin, L. *et al*. ydrochemical composition, distribution, and sources of typical organic pollutants and metals in Lake Bangong Co, Tibet. *Environ Sci Pollut Res* **28**, 9877-9888. <https://doi.org/10.1007/s11356-020-11449-w> (2020).
48. Buell, M. C., Johannessen, C., Drouillard, K. & Metcalfe, C. Concentrations and source identification of PAHs, alkyl-PAHs and other organic contaminants in sediments from a contaminated harbor in the Laurentian Great Lakes. *Environ Pollut* **270**, 116058. <https://doi.org/10.1016/j.envpol.2020.116058> (2021).
49. Zhang, Z. D. *et al*. A study on PAHs in the surface soil of the region around Qinghai Lake in the Tibet plateau: evaluation of distribution characteristics, sources and ecological risks. *Environ Res Commun* **3**, 041005. <https://doi.org/10.1088/2515-7620/abf3d9> (2021).
50. Lu L., Wang Z. & Pei J. G. Pollution characteristics and source analysis of polycyclic aromatic hydrocarbons in surface sediments from suburban type underground river in Nanning. *Environ Pollut* *Control* **42**, 597-603. (2020) (In Chinese).
51. Chen, C. F. *et al*. Distribution, sources, and behavior of PAHs in estuarine water systems exemplified by Salt River, Taiwan. *Mar Pollut Bull* **154**, 111029. <https://doi.org/10.1016/j.marpolbul.2020.111029> (2020).
52. Kumar, B., Verma, V. K., Kumar, S., Sharma, C. S. & Akolkar, A. B. Benzo(a)Pyrene equivalency and source identification of priority polycyclic aromatic hydrocarbons in surface sediments from Yamuna River. *Polycyclic Aromatic Compounds* **40**, 396-411. <https://doi.org/10.1080/10406638.2018.1441878> (2020).
53. He, S. H. *et al*. Distribution,source and ecological risk assessment of polycyclic aromatic hydrocarbons (PAHs) in surface sediments from Sanya River. *Environ* *Chem* **38**, 967-970. (2019) (In Chinese).
54. Zhang, J. M., Huang, H., Wang, R. J. & Sun, R. Y. Historical Pollution and Source Contributions of PAHs in Sediment Cores from the Middle Reach of Huai River, China. *Bull Environ Contam Toxicol* **102**, 531-537. <https://doi.org/10.1007/s00128-019-02576-3> (2019).
55. Yang, Y. Y. *et al*. Distributions, compositions, and ecological risk assessment of polycyclic aromatic hydrocarbons and phthalic acid esters in surface sediment of Songhua river, China. *Mar Pollut Bull* **152**, 110923. <https://doi.org/10.1016/j.marpolbul.2020.110923> (2020).
56. Du, S. L. *et al.* Pollution and ecological risk assessment of polycyclic aromatic hydrocarbons in the water environment of Shaying River Basin, China. *Nong Ye Huan Jing Ke Xue Xue Bao* **39**, 601-611. (2020) (In Chinese).
57. Sogbanmu, T. O., Osibona, A. O. & Otitoloju, A. A. Specific polycyclic aromatic hydrocarbons identified as ecological risk factors in the Lagos lagoon, Nigeria. *Environ Pollut* **255**, 113295. <https://doi.org/10.1016/j.envpol.2019.113295> (2019).
58. Seo, S. H., Kwon, H. O., Park, M. K., Lee, I. S. & Choi, S. D. Contamination characteristics of polycyclic aromatic hydrocarbons in river and coastal sediments collected from the multi-industrial city of Ulsan, South Korea. *Mar Pollut Bull* **160**, 111666. <https://doi.org/10.1016/j.marpolbul.2020.111666> (2020).
59. Xie, Z. L. *et al*. Characteristics, sources, and risks of polycyclic aromatic hydrocarbons in topsoil and surface water from the Liuxi River Basin, South China. *Arch Environ Contam Toxicol* **78**, 401-415. <https://doi.org/10.1007/s00244-020-00711-4> (2020).
60. Benson, N.U. *et al*. Occurrence, depth distribution and risk assessment of PAHs and PCBs in sediment cores of Lagos lagoon, Nigeria. *Reg Stud Mar Sci* **37**, 101335. <https://doi.org/10.1016/j.rsma.2020.101335> (2020).
61. Toan, V. D., Quynh, T.X. & Huong, N. T. L. Endocrine disrupting compounds in sediment from KimNguu river, Northern area of Vietnam: a comprehensive assessment of seasonal variation, accumulation pattern and ecological risk. *Environmental Geochemistry and Health* **42**, 647-659. <https://doi.org/10.1007/s10653-019-00399-z> (2020).
62. Deng, Q. C. *et al*. Sedimentary evolution of PAHs, POPs and ECs: Historical sedimentary deposition and evolution of persistent and emerging organic pollutants in sediments in a typical karstic river basin. *Sci Total Environ* **773**, 144765. <https://doi.org/10.1016/j.scitotenv.2020.144765> (2021).
63. Stakeniene, R., Joksas, K., Galkus, A. & Raudonyte-Svirbutaviciene, E. Polycyclic aromatic hydrocarbons in surface sediments from the Curonian Lagoon and the Nemunas River Delta (Lithuania, Baltic Sea): distribution, origin, and suggestions for the monitoring program. *Environ Monit Assess* **191**, 212. <https://doi.org/10.1007/s10661-019-7367-6> (2019).
